# Detection of *Chilomastix mesnili* in Common Marmoset (*Callithrix jacchus*) and Treatment with Metronidazole

**Published:** 2019

**Authors:** Eui-Suk JEONG, Jong-Hyung PARK, Seung-Hyun RYU, Soo-Young CHOI, Kyoung-Sun LEE, Jong-Man KIM, Byung-Hwa HYUN, Yang-Kyu CHOI

**Affiliations:** 1. Department of Laboratory Animal Medicine, College of Veterinary Medicine, Konkuk University, Seoul 05029, Republic of Korea; 2. Laboratory Animal Center, Daegu-Gyeongbuk Medical Innovation Foundation, Daegu 41061, Republic of Korea; 3. Laboratory Animal Center, Osong Medical Innovation Foundation, Chungbuk 28160, Republic of Korea

**Keywords:** *Chilomastix mesnili*, Common marmoset, *Escherichia coli*, Metronidazole, *Proteus mirabilis*

## Abstract

**Background:**

Recently, the use of common marmoset (*Callithrix jacchus*) has increased in biomedical research as an animal model. This study aimed to test fecal samples to monitor bacterial and parasite infections in common marmoset at the Laboratory Animal Center of Osong Medical Innovation Foundation in Korea.

**Methods:**

To monitor bacteria and parasites in common marmoset, we tested 43 fecal samples of 43 common marmosets by culture and parasitological test in 2014. Infection by *Chilomastix mesnili* was determined by PCR method.

**Results:**

We identified nonpathogenic bacteria such as *Proteus mirabilis* and *Escherichia coli* in feces of normal common marmosets. Interestingly, *C. mesnili* was isolated from a healthy common marmoset by fecal centrifugation concentration and PCR. The monkey infected with *C. mesnili* was treated with metronidazole. After the treatment, *C. mesnili* were not found in feces using fecal centrifugation concentration and PCR.

**Conclusion:**

This is the first case report of *C. mesnili* infection in common marmoset. Treatment with metronidazole is found to be highly effective in eradicating *C. mesnili* infection in common marmoset.

## Introduction

Non-human primates (NHPs) are excellent experimental models for biomedical research such as physiology, anatomy, immunology, and neurology. The key advantage of using NHPs is that they share great genetic and physiological similarities with humans. Recently, the use of common marmoset (*Callithrix jacchus*) has increased in biomedical research as an animal model. Common marmoset is a member of the New World monkeys (1). Compared to rhesus monkey (*Macaca mulatta*) or cynomolgus monkey (*M. fascicularis*), common marmoset has several advantages as an animal model because it is small (adult: 350–400 g) and typically produces twins or triplets (1). Furthermore, it is easy to handle. Therefore, it is an economically attractive NHP species for biomedical research.

Animal experiments are essential to progress in biomedical science. Health surveillance is important for infection prevention and defining pathogen status. Colonies of research animals are susceptible to infection with a variety of pathogenic bacterial, viruses, and parasites (2, 3). In many instances, these agents produce no clinical signs. However, they result in physiologic changes that may alter, and in some cases, invalidate research carried out with infected animals (2–4). In addition, some zoonotic pathogens may infect researcher-using animals. Therefore, it is important to keep laboratory animals free from infectious pathogens (2, 3)

Over the past several decades, the health status of experimental animals has greatly improved because of resulting in increased availability of animals free of infectious agents. Health monitoring refers to the practice of regular and repeated inspection of laboratory animals to determine whether pathogens are infected (2). A program of health monitoring is essential to assure managers and researchers that the standards of animal health within the facility are maintained so that experiments will not be compromised by unexpected infections. In this study, we tested fecal samples to monitor bacterial and parasite infections in common marmoset.

## Materials and Methods

### Specimens

Total 43 fresh fecal samples of 43 common marmosets (*C. jacchus*) were obtained from the Laboratory Animal Center of Osong Medical Innovation Foundation (Chungbuk, Republic of Korea) in 2014. All common marmosets were housed in stainless steel cage. There were maintained in 12 h dawn and 12 h dusk conditions at a temperature of 24 °C and humidity 40% to 60% in the SPF animal facility. Common marmosets were provided with a standard marmoset diet (Clea New World Monkey Diet, CMS-1M; CLEA Japan Inc., Tokyo, Japan) and water *ad libitum*.

All protocols were approved by the Committee on Animal Care of the Laboratory Animal Center of Osong Medical Innovation Foundation.

### Culture tests

Culture tests were performed to isolate *Campylobacter jejuni, Salmonella* spp., *Shigella* spp., *Staphylococcus aureus,* and *Yersinia pseudotuberculosis*. Fecal contents were streaked onto selective agars such as Campy-BAP (BD Diagnostics, MD, USA), Cefsulodin-Irgasan-Novobiocin (CIN, BD Diagnostics), DHL (Merck Millipore Corporation, Darmstadt, Germany), Salmonella-Shigella (SS, BD Diagnostics), and Volgel-Johnson (BD Diagnostics). After incubation of the agar plates at 37 °C for 24 or 48 h, colonies were recovered. We used the biochemical test and VITEK® 2 compact automated systems (bio-Mérieux, Inc., Marcy l’Etoile, France).

### Parasitological tests

Parasitological tests were performed to isolate intestinal helminths and protozoa. Parasitological tests were used fecal flotation and fecal centrifugation concentration (FCC) by zinc sulfate (specific gravity: 1.18) method (5). We differentiated *Entamoeba histolytica,* G*iardia* spp*.* etc. by protozoal movement patterns and morphology.

### Giemsa stain

Giemsa stain was used in histopathological diagnosis of intestinal helminths and protozoa. A thin film of the specimen on a microscope slide was fixed in methanol for 5 min. The slide was immersed in a freshly prepared 20% Giemsa stain solution (Sigma-Aldrich, Inc., MO, USA) for 10 min, then rinse slides with tap water, air dry and evaluate.

### PCR amplifications

Fecal DNA was extracted using an AccuPrep genomic DNA extraction kit (Bioneer, Daejeon, Republic of Korea). PCR was performed to identify intestinal protozoa. The *Chilomastix mesnili* specific PCR primers 5′ GCA GTT CTT TCG TGA TTG TGA 3′ and 5′ GAG GTC TCG TCC GTT ATC G 3′ designed to small subunit ribosomal RNA gene (94 bp, GenBank accession number EU009465). For PCR, DNA was denatured at 95 °C for 15 sec, annealed at 60 °C for 10 sec, and extended at 72 °C for 30 sec. This process was repeated for 35 cycles. Detection for *Giardia intestinalis, Entamoeba histolytica,* and *Pentatrichomonas hominis* were performed with primers described by previous reports (6).

PCR amplification was performed to differentiate *Helicobacter* spp. We used species-specific PCR primer 5′ CTA TGA CGG GTA TCC GGC 3′ and 5′ ATT CCA CCT ACC TCT CCC A 3′ designed to 16S ribosomal RNA gene sequence (7). For PCR, DNA was denatured at 94 °C for 2 sec, annealed at 53 °C for 2 sec, and extended at 72 °C for 30 sec. This process was repeated for 35 cycles. All PCR products were separated on a 1.8% agarose gel and stained with ethidium bromide.

### Metronidazole treatment

Metronidazole is on the WHO’s List of Essential Medicines, a list of the most important medication needed in a basic health system. The monkey infected with *C. mesnili* was treated orally with metronidazole (30 mg/kg body weight) for 1 week (daily, 3 times), immediately after detection of parasite infection.

## Results

### Bacteria detection in feces

**Fig. 1:**
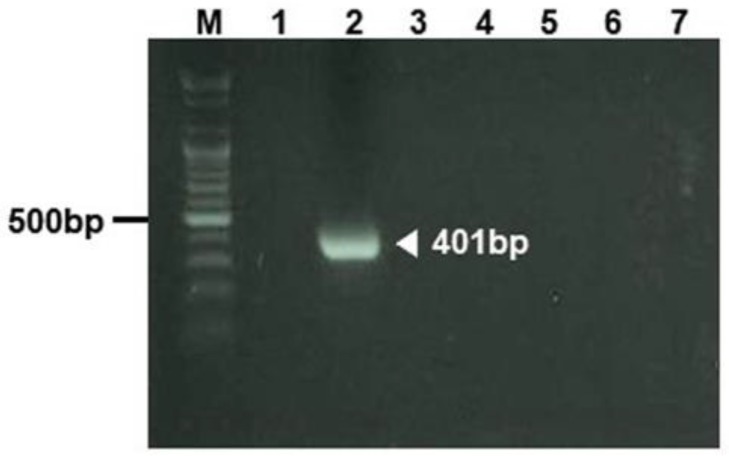
*Helicobacter* genus-specific DNA amplified by PCR in fecal sample. M; marker, lane 1; negative control, lane 2; positive control, lanes 3 and 7; fecal samples collected from common marmoset number 1 to 5

To monitor pathogenic bacteria such as *Campylobacter jejuni, Helicobacter* spp., *Salmonella* spp., *Shigella* spp., *S. aureus,* and *Yersinia pseudotuberculosis*, we tested 43 fecal samples of 43 common marmosets. We used culture test to detect pathogenic bacteria except for *Helicobacter* spp. To detect *Helicobacter* spp. in feces, PCR method was used because of the most efficient and fast method. Pathogenic bacteria were not identified in all feces from 43 common marmosets (Table 1 and Fig. 1). However, nonpathogenic bacterium *Proteus mirabilis* was isolated from 4 common marmosets. Prevalence of *P. mirabilis* was approximately 9.3%. Nonpathogenic *Escherichia coli* was isolated from most common marmosets.

**Table 1:** Prevalence of bacteria in common marmosets. Culture and PCR tests were performed

***Organism***	***Sample Tested***	***Test Method***	***Result***
*Campylobacter jejuni*	Feces	Culture	0/43
*Helicobacter* spp.	Feces	PCR	0/43
Nonpathogenic *E. coli*	Feces	Culture	31/43
*Proteus mirabilis*	Feces	Culture	4/43
*Salmonella* spp.	Feces	Culture	0/43
*Shigella* spp.	Feces	Culture	0/43
*Staphylococcus aureus*	Feces	Culture	0/43
*Yersinia pseudotuberculosis*	Feces	Culture	0/43

### Parasite detection in feces

**Fig. 2:**
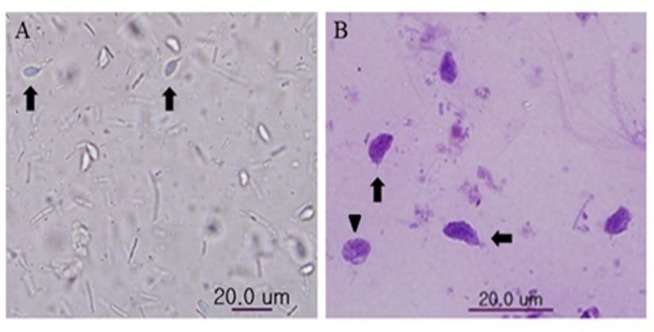
*C. mesnili* was isolated from a common marmoset by fecal centrifugation concentration technique. Trophozoite (arrow) and cyst (arrowhead) of *C. mesnili* from fecal sample (A) and stained with Giemsa stain (B).

**Fig. 3:**
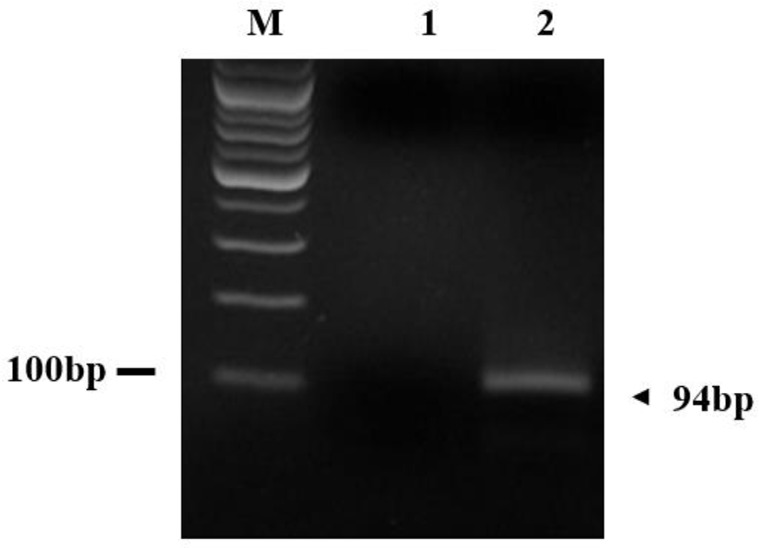
*C. mesnili* –specific DNA amplified by PCR in fecal sample. M; marker, lane 1; negative control, lane 2; fecal sample collected from common marmoset number 5

To monitor eggs of intestinal helminths and protozoa, we tested 43 fecal samples of 43 common marmosets using fecal flotation and fecal centrifugation concentration (FCC) method. Intestinal protozoa *C. mesnili* was isolated from one common marmoset (animal number 5). Prevalence of *C. mesnili* was approximately 2.3%. Morphology of *C. mesnili* was observed by Giemsa stain (Fig. 2). The identification of *C. mesnili* was also confirmed by PCR (Fig. 3).

To remove protozoa, the common marmoset infected with *C. mesnili* was treated with metronidazole for one week. We monitored the common marmoset infected with *C. mesnili* every 3 months for 1.5 yr using FCC and PCR. After 1.5 yr, cysts, trophozoites, or PCR products of *C. mesnili* were not found in the feces. Therefore, *C. mesnili* was eradicated from this common marmoset

## Discussion

We identified nonpathogenic bacteria such as *P. mirabilis* and *E. coli* in feces of common marmosets. In addition, *C. mesnili* was isolated from a healthy common marmoset by FCC technique and PCR. This is the first reported case of *C. mesnili* infection in a specific-pathogen-free common marmoset. The monkey infected with *C. mesnili* was treated orally with metronidazole. After treatment with metronidazole for one week, *C. mesnili* was not found in the feces anymore. *P. mirabilis* is a Gram-negative bacterium with swarming colony and urease activity. It is capable of causing human infection. Its infections have been reported in dog and human (8). *However, it is not* on the pathogenic bacteria list of New World monkey or Old World monkey (4).

*C. mesnili* is one of the zoonotic intestinal protozoa in Korea*.* In 2250 samples collected from 1969 to 1970 in the Republic of Korea, 0.5% of samples have been infected by *C. mesnili* (9). The prevalence of *C. mesnili* infection has been reported to be 21% in non-human primates of four zoological gardens in Belgium. However, New World monkeys including tamarin, marmoset, spider monkey, and squirrel monkey are not infected by *C. mesnili* in Belgium (10). Recently, the infectious rate of *C. mesnili* is reported to be 5.2% in wild chimpanzees (*Pan troglodytes troglodytes*) in southeast Cameroon (11). *C. mesnili* is not on the pathogenic protozoa list of New World monkey or Old World monkey (4). Even though *C. mesnili* is a commensal non-pathogenic protozoan, it is important to keep laboratory monkey free from parasites because of improved health and longevity of marmosets in SPF facility (12). To the best of our knowledge, this study is the first one that reports *C. mesnili* infection in common marmoset.

In the present study, we successfully detected small subunit ribosomal RNA gene of *C. mesnili* among intestinal protozoa in feces by PCR. PCR analysis has been used for the detection and identification of intestinal protozoa in fecal samples of human (6) and new world monkeys (13). Interestingly, *Pentatrichomonas hominis* has been found in both normal feces and diarrheal feces of laboratory-bred common marmosets (13). In this study, a common marmoset infected with *C. mesnili* was treated orally with metronidazole. Metronidazole is widely used to treat infections of protozoa in cats (14) and monkeys (15). We examined *C. mesnili* infection by FCC and PCR every 3 months for 1.5 year. Cysts, trophozoites, or PCR product of *C. mesnili* were not found in the feces after one-week treatment with metronidazole.

## Conclusion

*C. mesnili* was isolated from a healthy common marmoset at the Laboratory Animal Center of Osong Medical Innovation Foundation by fecal centrifugation concentration and PCR*.* This is the first reported case of *C. mesnili* infection in common marmoset. Treatment with metronidazole was highly effective to eradicate *C. mesnili* infection.
